# Effects of the *ABCB1* (1199G > A) Polymorphism on Steroid Sex Hormone-Induced P-Glycoprotein Expression, ATPase Activity, and Hormone Efflux

**DOI:** 10.3390/medsci3040124

**Published:** 2015-12-01

**Authors:** Rui Peng, Hong Zhang, Ying Zhang, Dan-Yun Wei

**Affiliations:** Department of Pharmacy, Renmin Hospital of Wuhan University, Wuhan 430060, China; E-Mails: pengruiwec@hotmail.com (R.P.); zhangdezhou@hotmail.com (Y.Z.); weiwhuhan@hotmail.com (D.-Y.W.)

**Keywords:** *ABCB1* (1199G > A), polymorphism, P-gp, steroid sex hormone, transport

## Abstract

This study examined how the 1199G > A polymorphism in the *ABCB1* gene encoding P-glycoprotein (P-gp) affects the protein’s expression, ATPase activity, and ability to pump female steroid sex hormones out of LLC-PK1 cells. The *ABCB1* (1199G) or *ABCB1* (1199A) allele was transfected into cells, which were incubated for 48 h with various hormone concentrations, then analyzed by Western blotting to examine expression of P-gp protein and by reverse transcription-polymerase chain reaction (RT-PCR) to examine expression of mRNA. Cells were also compared in terms of their transepithelial permeability to steroid sex hormones in the presence and absence of the specific P-gp inhibitor GF120918. P-gp ATPase activity induced by steroid sex hormones was also assayed. Estriol and ethynyl estradiol up-regulated levels of *ABCB1* mRNA in a concentration-dependent manner, with *ABCB1* (1199A) mRNA showing greater up-regulation than *ABCB1* (1199G) mRNA. Estrone, estriol, and ethynyl estradiol were substrates of both types of P-gp in transepithelial permeability assays, and the *ABCB1* (1199A) protein showed a significantly higher net efflux ratio for estrone (13.4 *vs.* 7.4, *p* < 0.005), estriol (5.6 *vs.* 3.3, *p* < 0.05), and ethynyl estradiol (12.7 *vs.* 5.3, *p* < 0.005). Induction of P-gp ATPase activity by ethynyl estradiol and progesterone increased with increasing hormone concentration, and the magnitude of stimulation was greater for *ABCB1* (1199A) P-gp than for *ABCB1* (1199G) P-gp. These results indicate that the *ABCB1* (1199G > A) polymorphism influences steroid sex hormone-induced expression and function of P-gp, which may help to explain inter-patient differences in P-gp-mediated chemotherapy resistance *in vivo*.

## 1. Introduction

P-glycoprotein (P-gp), a transporter relation to multidrug resistance, is encoded by the human multidrug resistance gene (*ABCB1*, *MDR1*). P-gp localizes at the plasma membrane of both normal cells and tumor cells, where it mediates ATP-dependent efflux of various endogenous substances and lipophilic xenobiotics [1,2]. P-gp expression and activity vary substantially in the population, leading to significant differences in the pharmacokinetics and efficacy of chemotherapeutics. This variability in P-gp likely reflects, at least in part, genetic variability in the *ABCB1* gene. More than 60 single-nucleotide polymorphisms (SNPs) have been identified in *ABCB1*, including several in the protein-coding region (rs1128503, 1236C > T; rs2032582, 2677G > T/A; rs1045642, 3435C > T) [3,4]. Understanding the influence of the reported SNPs on P-gp expression and activity may help in overcoming multidrug resistance and in compensating for inter-patient differences in chemotherapeutic efficacy through personalized medicine.

While many studies have evaluated the influence of *ABCB1* polymorphisms on P-gp-mediated transport, results have not always been consistent [5]. In addition, that previous work has neglected some *ABCB1* SNPs, such as *ABCB1* (1199G > A), which leads to a Ser400Asn substitution in the encoded protein and which has an allelic frequency of approximately 6% among Caucasians [3,4,6]. The 1199G > A SNP may help predict the outcomes of paclitaxel chemotherapy in cancer patients, and the *ABCB1* (1199A) allele correlates with greater resistance to anti-prostate cancer therapy than the *ABCB1* (1199G) allele [7]. Cells expressing the *ABCB1* (1199A) allele show lower P-gp-mediated efflux of Rhodamine 123 than cells transfected with the *ABCB1* (1199A) allele [8]; the two types of cells display similar response to doxorubicin, while cells expressing the *ABCB1* (1199A) allele show stronger resistance to vinblastine and vincristine.

Little is known about how many of these SNPs, including 1199G > A, affect the responsiveness of P-gp to steroid sex hormones. In rat intestine, progesterone and other steroid hormones up-regulate P-gp expression and activity; in this way, progesterone reduces intestinal absorption of antitumor drug vinblastine, and perhaps the absorption of other substrates as well [9]. Progesterone, as well as several of its natural metabolites, are potent P-gp inhibitors *in vitro* [10]. Steroid sex hormones may modulate P-gp in a broad range of tissues, given that the protein is highly expressed in placenta, liver canalicular membranes, the blood-brain-barrier (BBB), intestinal mucosa, and kidney proximal tubules. Thus, P-gp is involved in the transport of several adrenal steroids, including cortisol, aldosterone, and dexamethasone [10,11,12]. More recently, *in vitro* studies have shown that estrone, estriol, and ethynyl estradiol are P-gp substrates that also up-regulate *ABCB1* transcription [13]. Steroid sex hormone-induced expression of P-gp may be important in normal human physiology: up-regulation of P-gp mRNA and protein in the endometrium varies across the menstrual cycle [14]. Such steroid sex hormone regulation is likely to be important for human chemotherapy, given that it may strongly influence the efficacy of orally-administered synthetic derivatives of estrogens and progestins.

Therefore, the present study examined whether the *ABCB1* 1199G > A polymorphism affects the ability of P-gp to recognize steroid sex hormones, such as estrogen and progesterone, as substrates, as well as the ability of such hormones to up-regulate P-gp expression and activity.

## 2. Materials and Methods

### 2.1. Materials

17β-Estradiol, ethynyl estradiol, estrone, estriol, progesterone, and norethindrone were obtained from Novartis Pharma AG (Basel, Switzerland). GF120918 was purchased from Selleck Chemicals (Houston, TX, USA). Stock solutions of all agents were dissolved in DMSO. Expression plasmids in which wild-type (1199G) or variant (1199A) cDNAs were subcloned into pcDNA 3.1 were obtained from Wuhan Biobuffer Biotech Service (Wuhan, China). RPMI 1640 medium, obtained from Thermo Fisher Scientific (Waltham, MA, USA), was mixed with 10% (*v*/*v*) fetal bovin serum (FBS; Thermo Fisher Scientific) and 1% (*v*/*v*) antibiotic-antimycotic solution (A-A; Thermo Fisher Scientific). Monoclonal antibody against P-gp was purchased from Signet (Dedham, MA, USA). Human P-gp and control membranes for ATPase activity assays were obtained from Gentest (Wuhan, China).

### 2.2. Construction of Recombinant LLC-PK1 Cell Lines

LLC-PK1 cell lines expressing the *ABCB1* (1199G) or *ABCB1* (1199A) allele were constructed by the Wuhan Biobuffer Biotech Service (Wuhan, China). Briefly, cells were plated in 25-cm culture dishes (6–8 × 10^6^ cells/dish) and transfected with 10 μg of expression plasmids *ABCB1*_1199G_ or *ABCB1*_1199A_ using Lenti-Pac™ (GeneCopoeia, Rockville, MD, USA) based on the manufacturer’s instruction. After transfection, cells were cultured in six-well plates (1.0 × 10^6^ cells/well) in the presence of 8 μg/mL puromycin to select for stable transfectants. In subsequent experiments, stably transfected cultures were maintained in six-well plates (1.0 × 10^6^ cells/well) in the presence of 2 μg/mL puromycin.

### 2.3. Cell Culture

Stably transfected *ABCB1* (1199G)-LLC-PK1 and *ABCB1* (1199A)-LLC-PK1 cells, as well as untransfected LLC-PK1 cells, were grown in six-well plates (3 × 10^5^ cells) in medium mixed with FBS and A-A. Transfected cultures were cultured in the presence of 2 μg/mL puromycin to select for cells overexpressing P-gp. All three types of cultures were then incubated in the presence or absence of the indicated steroid sex hormones at 37 °C in a moderately humid atmosphere of 5% CO_2_. Before bidirectional transport studies, transepithelial electrical resistance (TEER) was measured in each well to confirm the confluence and integrity of the cell monolayer; values ranged from 330 to 400 Ω/cm^2^ across the three types of cultures.

### 2.4. Flow Cytometry

P-gp expression on the surface of stably-transfected *ABCB1* (1199G)-LLC-PK1 and *ABCB1* (1199A)-LLC-PK1 cells was determined by flow cytometry as described. Briefly, LLC-PK1 cells were washed twice with ice-cold, 0.22 μm-filtered Hank's buffer containing 3% decomplemented FBS and 20 mM NaN_3_ (HAFA), then immobilized using 1% formaldehyde for 15 min at room temperature. Cells were rinsed three times with HAFA, then incubated in HAFA containing rabbit anti-P-gp monoclonal antibody (1:10; Abcam, AB129450) for 45 min on ice in the dark. Cells were again washed twice with HAFA and incubated with FITC-conjugated goat anti-rabbit IgG (1:50; Abcam, AB6717) for 30 min on ice in the dark. Cells were re-suspended in HAFA and analyzed by flow cytometry on a FACSCanto II device (BD Biosciences, San Jose, CA, USA).

### 2.5. Cell Lysis

*ABCB1* (1199G)-LLC-PK1 and *ABCB1* (1199A)-LLC-PK1 cell lines were grown for 48 h in six-well plates in RPMI 1640 supplemented with various concentrations of steroid sex hormones. Then cells were washed twice in ice-cold phosphate-buffered saline (PBS) and lysed in buffer containing 1 μg/mL pepstatin, 20 μg/mL leupeptin, 20 μg/mL aprotinin, 1 mM PMSF, and a protease inhibitor cocktail in hypotonic buffer (Tris-HCl, KCl, MgCl_2_). The cell lysate was stored at −80 °C.

### 2.6. Western Blot Analysis

Protein (40 μg) was fractionated on 8% polyacrylamide-SDS gels, transferred to nitrocellulose membranes and sealed for 1 h in nonfat dried milk in TBST. Membranes were probed first with rabbit anti-P-gp monoclonal antibody (1:3000; Abcam, AB129450), followed by HR dye-conjugated goat anti-rabbit IgG (1:10,000; Abcam, AB6532); both incubations were for 1 h at room temperature. Antibody binding was detected, and band intensity quantified, using the Odyssey IR imaging system (LI-COR Biosciences, Lincoln, NE, USA).

### 2.7. RT-PCR

*ABCB1* (1199G)-LLC-PK1 and *ABCB1* (1199A)-LLC-PK1 cell lines were cultured in six-well dishes (1.0 × 10^6^ cells/well), and total RNA was extracted using TRIZOL (Fermentas, Walthma, MA, USA). Purity and concentration of RNA extracts were checked using a UV spectrophotometer; extracts were used only if the absorbance ratio (260/280 nm) was 1.8–2.0. Extracts were diluted and stored at −80 °C until they were used to generate cDNA by reverse transcription RT-PCR as described. RT-PCR was performed on the Prism 7700 Sequence Detection System (ABI, Foster City, CA, USA), and it involved one step at 50 °C for 2 min, one step at 95 °C for 10 min, and then 40 cycles at 95 °C for 30 s and 60 °C for 30 s. The sense *ABCB1* primer was 5′-ATGTTTCCGGTTTGGAGCCT-3′; the anti-sense primer, 5′-TCCTTCCAATGTGTTCGGCA-3′. SYBR Green/Fluorescein qPCR Master Mix (2X; Fermentas, catalog No. ko242) was used. Levels of *ABCB1* mRNA were normalized to levels of β-actin transcript.

### 2.8. Transepithelial Permeability Assay

Bidirectional transport across monolayers of *ABCB1* (1199G)-LLC-PK1, *ABCB1* (1199A)-LLC-PK1, and untransfected LLC-PK1 cells were assayed as described [15], with a slight modification. Cells were plated in six-well Transwell dishes with inserts and TEER was measured as described in Section 2.3. Then, cells were washed with Hank’s Balanced Salt Solution containing 1% FBS and 22.5 mM HEPES (pH 7.4) (HBSS-GH) and incubated with shaking for another 30 min at 37 °C. Transport of potential P-gp substrates (estrone, estriol, and ethynyl estradiol) from the basolateral to apical side of the insert was measured by adding various concentrations of substrate to the basolateral chamber and HBSS-GH to the apical chamber. Cells were incubated with shaking at 37 °C, and 200-μL isometric medium were removed from the opposite side at 0.5, 1, 1.5, and 2 h. The analogous procedure was followed to measure transport from the apical to basolateral side of the insert. After sampling, 200 μL of fresh medium was added to the appropriate chamber to maintain the same initial volume. In some experiments, the HBSS-GH in both chambers was supplemented with 1.0 μM GF120918 to inhibit P-gp-mediated transport.

Steroid sex hormones in the harvested HBSS-GH were extracted with organic solvent, evaporated to dryness and redissolved. The supernatant was analyzed using high-performance liquid chromatography (HPLC). Apparent permeability (P_app_) was obtained from the equation *P*_app_ = (∆*Ǫ*/∆*t*)/(*C*_0_ × *A*), where ∆*Ǫ*/∆*t* is the linear rate of accumulation of steroid sex hormone in the receiver chamber, *C*_0_ represents the initial concentration of hormone, and *A* is the surface area of the osmotic membrane. Directionality of transport was quantified by calculating the net efflux ratio, defined as the ratio *P*_app_(basolateral → apical)/*P*_app_(apical → basolateral). The minimum efflux ratio for P-gp-mediated efflux was defined to be 2.0.

### 2.9. P-gp ATPase Activity Assay

Membrane fractions were prepared and evaluated for P-gp ATPase activity as described [16], with slight modifications. In parallel, human P-gp and control membranes (Gentest) were also evaluated. Membranes were incubated at 37 °C for 20 min in the presence of buffer (50 mM Tris-MES, 50 mM KCl, 2 mM dithiothreitol, 3 mM MgATP, 2 mM EGTA, 5 mM sodium azide) supplemented with 20 μM verapamil (positive control) or increasing concentrations of steroid sex hormones. The same reactions were set up in parallel in the presence of the ATPase inhibitor sodium orthovanadate (100 μM) as a negative control. 10% SDS was added to the solution in order to terminate the reactions, after which zinc acetate and ammonium molybdate were added to final respective concentrations of 15 and 35 mM. Over the next 20 min, absorbance at 800 nm was continuously monitored at room temperature to track the release of inorganic phosphate. Phosphate amounts were quantified against a phosphate standard curve.

## 3. Results

### 3.1. P-gp Expression in LLC-PK1 Cells Expressing ABCB1 (1199G) or ABCB1 (1199A) Alleles

**Figure 1:**
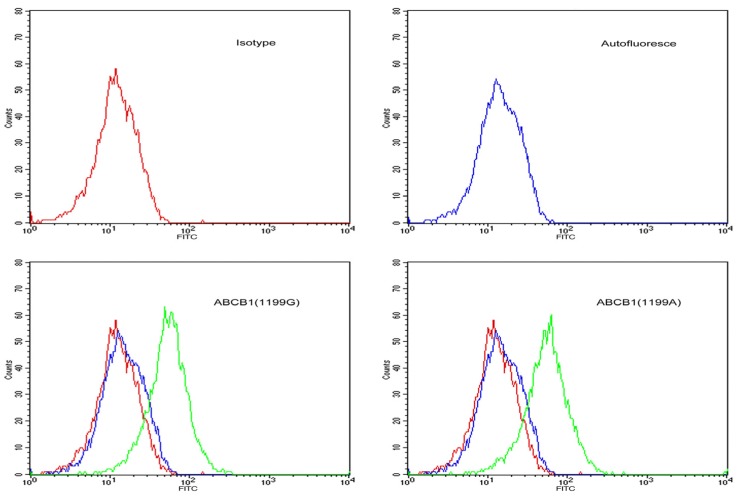
Flow cytometry histograms showing surface expression of P-gp in stably-transfected LLC-PK1 cells expressing the *ABCB1* (1199G) or *ABCB1* (1199A) allele of P-gp. Untransfected LLC-PK1 cells were also examined as a control. Cells were labelled with FITC-conjugated antibody against P-gp (green line), a matched isotypic control (red line), or left unlabelled (blue line).

LLC-PK1 cells expressing *ABCB1* (1199G) or *ABCB1* (1199A) alleles were incubated with FITC-conjugated goat anti-rabbit IgG against P-gp and analyzed by flow cytometry (Figure 1). Median fluorescence intensity was 97 arbitrary units in *ABCB1* (1199G) cells and 105 arbitrary units in *ABCB1* (1199A) cells, suggesting similar surface expression of P-gp. In contrast, untransfected LLC-PK1 cells showed weak fluorescence signal, suggesting negligible P-gp surface expression.

### 3.2. Steroid Sex Hormone-Induced Expression of P-gp Protein

**Figure 2:**
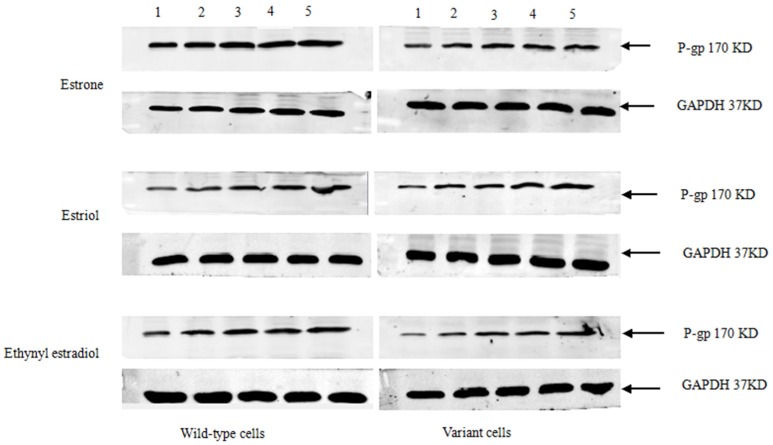
Western blotting of P-gp in LLC-PK1 cells expressing the *ABCB1* (1199G) or *ABCB1* (1199A) allele. Subconfluent cultures in complete medium were left untreated (lane 1) or treated with various concentrations (μM) of estrone, estriol, or ethynyl estradiol: lane 2, 0.1; lane 3, 0.2; lane 4, 0.4; lane 5, 0.8. Histograms show the inducible expression of P-gp in transfected cells in the presence of various concentrations of estrone, estriol, and ethynyl estradiol.

Stably-transfected LLC-PK1 cells expressing the *ABCB1* (1199G) or *ABCB1* (1199A) allele of P-gp, together with untransfected LLC-PK1 cells as a control, were treated for 48 h with various concentrations of estrone, estriol, and ethynyl estradiol (Figure 2). Cells were lysed and fractionated by SDS-PAGE, then analyzed by Western blotting to examine P-gp levels. All three sex hormones up-regulated the levels of P-gp. In contrast, progesterone, estradiol, or norethisterone did not significantly affect levels of either type of P-gp (data not shown).

### 3.3. Steroid Sex Hormone-Induced Expression of P-gp mRNA

**Figure 3:**
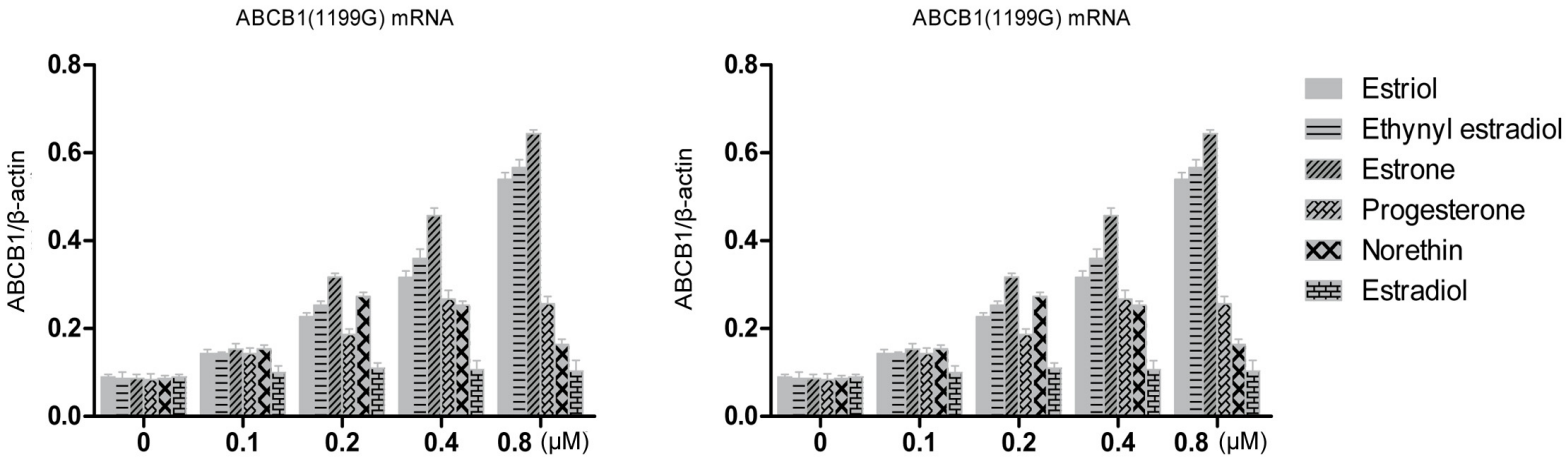
RT-PCR quantitation of P-gp transcripts expressed by LLC-PK1 cells expressing the *ABCB1* (1199G) or *ABCB1* (1199A) allele, following treatment with steroid sex hormones (0.1–0.8 μM). Results for different hormones at the same concentration are colored the same.

Stably-transfected LLC-PK1 cells expressing the *ABCB1* (1199G) or *ABCB1* (1199A) allele of P-gp, together with untransfected LLC-PK1 cells as a control, were treated for 48 h with various concentrations of estrogens and progestins. Then total cellular RNA was extracted and levels of P-pg mRNA were quantified using RT-PCR (Figure 3). All three hormones up-regulated levels of both mRNAs in a concentration-dependent manner. At a concentration of 0.8 μM, estriol treatment up-regulated levels of *ABCB1* (1199G) mRNA 6.0-fold (over the control); ethynyl estradiol, 6.5-fold; and estrone, 7.4-fold. The corresponding fold increases in levels of *ABCB1* (1199A) mRNA were 10.3, 10.7 and 7.9. Statistical analysis showed that estriol and ethynyl estradiol up-regulated *ABCB1* (1199A) mRNA levels significantly more than *ABCB1* (1199G) mRNA levels (Table 1). In contrast, estrone up-regulated the two mRNAs to a similar extent.

**Table 1:** Quantitation of steroid sex hormone-induced up-regulation of *ABCB1* (1199G) and *ABCB1* (1199A) mRNAs.

Sex-Steroid Hormones	Concentrations	*ABCB1* (1199G) mRNA (Fold Induction, over Control)	*ABCB1* (1199A) mRNA (Fold Induction, over Control)
Estriol	0.1 μM	1.56 ± 0.02	3.00 ± 0.04 *
	0.2 μM	2.56 ± 0.03	4.25 ± 0.07 *
	0.4 μM	3.56 ± 0.09	6.88 ± 0.12 **
	0.8 μM	6.00 ± 0.11	10.25 ± 0.14 **
Ethynyl Estradiol	0.1 μM	1.61 ± 0.04	2.75 ± 0.06 *
	0.2 μM	2.87 ± 0.09	4.88 ± 0.12 **
	0.4 μM	4.14 ± 0.09	7.25 ± 0.05 **
	0.8 μM	6.55 ± 0.13	10.75 ± 0.20 **
Estrone	0.1 μM	1.72 ± 0.02	1.88 ± 0.03
	0.2 μM	3.68 ± 0.05	4.00 ± 0.07
	0.4 μM	5.29 ± 0.11	5.63 ± 0.09
	0.8 μM	7.90 ± 0.16	7.88 ± 0.11

Results are the mean ± SD of three replicates. * *p* < 0.05, ** *p* < 0.001 between the two mRNAs.

Norethindrone at 0.2 μM up-regulated *ABCB1* (1199G) mRNA 3.2-fold and *ABCB1* (1199A) mRNA 2.8-fold, which was greater than the up-regulation observed at higher concentrations. This may reflect variations in the amount of template cDNA. Progesterone at 0.4 μM up-regulated levels of both types of mRNA three-fold, while estradiol caused only slight increases in the level of either mRNA.

These results suggest that estrogens and progestins can regulate *ABCB1* transcription, and that this regulation is significantly affected by the 1199G > A polymorphism in the case of some hormones but not others.

### 3.4. Transepithelial Permeability of LLC-PK1 Monolayers to Steroid Sex Hormones in the Presence of ABCB1 (1199G)- or ABCB1 (1199A)-Encoded P-gp

**Figure 4:**
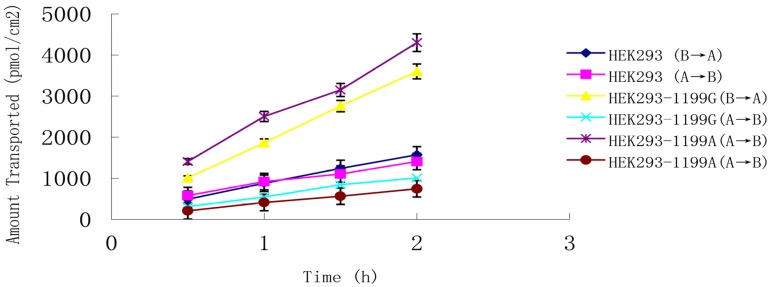
Transepithelial transport of 0.8 μM estriol across monolayers of LLC-PK1 cells expressing the *ABCB1* (1199G) or *ABCB1* (1199A) allele. Untransfected LLC-PK 1 cells served as a control. Estriol was added to the basolateral or apical chamber, and the medium in the opposite chamber was sampled at 0.5, 1.0, 1.5, and 2 h. Data shown are mean ± SD of three replicates.

Natural and synthetic estrogen and progesterone were evaluated to determine whether they can be transported by *ABCB1* (1199G) and *ABCB1* (1199A) P-gp across an LLC-PK1 cell monolayer system. Estriol at 0.8 μM was transported in both basolateral-to-apical and apical-to-basolateral directions (Figure 4), and *P*_app_ from the basolateral side was much higher than that from the apical side, giving a net efflux ratio of 3.3 for cells expressing *ABCB1* (1199G) P-gp and 5.6 for cells expressing *ABCB1* (1199A) P-gp (Table 2). The corresponding net efflux ratio was only 1.1 in untransfected cells. These results suggest that estriol is a good substrate for both types of P-gp.

Transport of estradiol and estrone was greater in cells expressing either type of P-gp than in control cells, and net efflux ratios were >2.0, suggesting that these hormones are also good substrates for the transporter (Table 2). The net efflux of ethynyl estradiol was significantly higher in cells expressing *ABCB1* (1199A) P-gp than in cells expressing *ABCB1* (1199G) P-gp (12.7 *vs.* 5.3, *p* < 0.005). The same was true for the net efflux of estrone (13.4 *vs.* 7.4, *p* < 0.005). These results suggest that the change from 1199G to A leads to significantly greater ability to transport ethynyl estradiol and estrone.

Net efflux ratios were much lower than two for progesterone, norethindrone, and estradiol. Thus, these hormones are unlikely to be P-gp subtrates.

**Table 2:** Transepithelial permeability of untransfected LLC-PK1 cells and LLC-PK1 cells expressing the *ABCB1* (1199G) or *ABCB1* (1199A) allele to hormones in the presence or absence of P-gp inhibitor GF120918.

Hormone	*P*_appB→A_/*P*_appA→B_ (SD)		
	LLC-PK1	*ABCB1* (1199G)-LLC-PK1	*ABCB1* (1199A)-LLC-PK1
Estriol (0.8 μM)	1.1 (0.2)	3.3 (0.8)	5.6 (1.1) *
Estriol + GF120918	1.3 (0.4)	0.9 (0.2)	1.1 (0.4)
Ethynyl estradiol (0.8 μM)	0.9 (0.2)	5.3 (2.4)	12.7 (3.1) **
Ethynyl estradiol + GF120918	1.3 (0.4)	1.4 (0.5)	0.8 (0.2)
Estrone (0.2 μM)	1.4 (0.3)	7.4 (1.4)	13.4 (4.1) **
Estrone + GF120918	1.6 (0.4)	1.8 (0.7)	1.5 (0.2)
Estradiol (0.6 μM)	0.5 (0.1)	1.2 (0.4)	1.7 (1.1)
Estradiol + GF120918	0.7 (0.2)	1.4 (0.5)	1.6 (0.4)
Progesterone (0.8 μM)	0.8 (0.3)	1.1 (0.1)	1.4 (0.2)
Progesterone + GF120918	0.9 (0.4)	1.0 (0.3)	1.2 (0.3)
Norethindrone (1.0 μM)	0.6 (0.2)	1.3 (0.4)	1.7 (0.2)
Norethindrone + GF120918	0.9 (0.3)	1.4 (0.5)	1.9 (0.2)

Values are mean ± SD of three replicates. * *p* < 0.05, ** *p* < 0.005 between cells expressing the *ABCB1* (1199G) or *ABCB1* (1199A) allele.

To confirm the results for ethynyl estradiol, estrone, and estriol, we re-measured net efflux ratios of these three hormones in the presence of the specific P-gp inhibitor GF120918 (1.0 μM). Addition of GF120918 to both apical and basolateral chambers completely inhibited efflux of all three hormones in cells expressing the *ABCB1* (1199G) or *ABCB1* (1199A) allele.

### 3.5. Steroid Sex Hormone-Induced ATPase Activity in P-Gp

**Figure 5:**
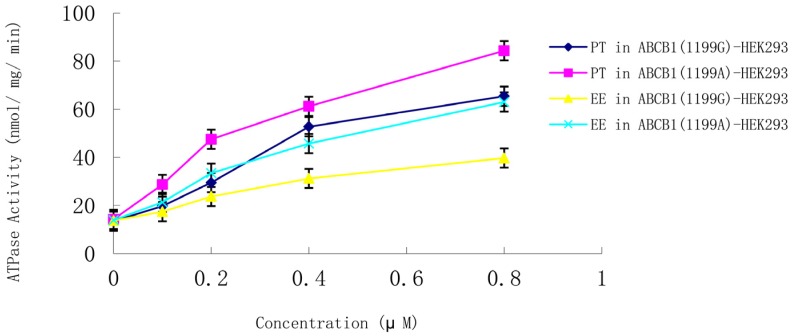
Concentration-dependent stimulation of P-gp ATPase activity by progesterone and ethynyl estradiol (0.1–0.8 μM) in membranes from LLC-PK1 cells expressing the *ABCB1* (1199G) or *ABCB1* (1199A) allele. Data shown are mean ± SD of three replicates. EE, ethynyl estradiol; PT, progesterone.

Membranes from LLC-PK1 cells expressing the *ABCB1* (1199G) or *ABCB1* (1199A) allele were used to examine whether steroid sex hormones affect P-gp ATPase activity. Both ethynyl estradiol and progesterone (0.1–0.8 μM) induced ATPase activity in a concentration-dependent manner in cells expressing the *ABCB1* (1199G) or *ABCB1* (1199A) allele (Figure 5). ATPase induction was much higher in cells expressing the *ABCB1* (1199A) allele. Control experiments in the presence of vanadate confirmed that the observed ATPase activity was due to P-gp. None of the other hormones appreciably stimulated P-gp ATPase activity.

P-gp ATPase activity in the absence of any hormones was similar between cells expressing the *ABCB1* (1199G) or *ABCB1* (1199A) allele. In the presence of progesterone or ethynyl estradiol, significantly greater ATPase activity was induced in cells expressing *ABCB1* (1199G) P-gp than in cells expressing *ABCB1* (1199A) P-gp (Table 3). These results suggest that the change from 1199G to A leads to significantly greater ATPase induction by ethynyl estradiol and progesterone.

**Table 3:** ATPase activity of *ABCB1* (1199G) or *ABCB1* (1199A) P-gp after induction with ethynyl estradiol or progesterone.

Sex-Steroid Hormones	Concentrations	Wild-Type P-gp ATPase Activity (Fold Induction, over Control)	Variant P-gp ATPase Activity (Fold Induction, over Control)
Progesterone	0.1 μM	1.47 ± 0.01	2.02 ± 0.04 *
	0.2 μM	2.20 ± 0.03	3.35 ± 0.02 **
	0.4 μM	3.93 ± 0.08	4.31 ± 0.05 *
	0.8 μM	4.88 ± 0.07	5.94 ± 0.14 **
Ethynyl Estradiol	0.1 μM	1.07 ± 0.04	1.53 ± 0.06 **
	0.2 μM	1.73 ± 0.08	2.40 ± 0.02 **
	0.4 μM	2.28 ± 0.07	3.29 ± 0.04 **
	0.8 μM	2.90 ± 0.05	4.53 ± 0.11 **

Results are the mean ± SD of three replicates. * *p* < 0.05, ** *p* < 0.005 between *ABCB1* (1199G) and *ABCB1* (1199A) P-gp.

## 4. Discussion

Flow cytometry experiments indicated that surface expression of P-gp was similar in LLC-PK1 cells transfected with either the *ABCB1* (1199G) or *ABCB1* (1199A) allele. This suggests that the results obtained in this study do not reflect differences in baseline P-gp expression between the two cell lines. As a result, we were able to compare steroid sex hormone net efflux for both *ABCB1* (1199G) and *ABCB1* (1199A) P-gp.

Earlier studies [17,18] reported that P-gp polymorphisms played an important role in the development of steroid resistance in children with nephrotic syndrome. Cho *et al*. [19] showed an influence of the G3435T polymorphism in the *ABCB1* gene on the therapeutic efficacy of corticoids in patients with Crohn’s disease. Here we provide evidence that the *ABCB1* 1199G > A polymorphism significantly affects how much steroid sex hormones up-regulate P-gp mRNA and protein, and how much they activate P-gp ATPase activity *in vitro*. However, the influence of *ABCB1* 1199G > A polymorphism on hormones up-regulation P-gp mRNA, protein and ATPase activity *in vivo* were not reported and need further investigations. These findings show that modulation of P-gp activity by steroid sex hormones may give rise to different pharmacokinetics and outcomes of chemotherapy in patients, depending on their genotype at the *ABCB1* 1199G > A polymorphism. Our results provide testable hypotheses for explaining why the *ABCB1* (1199A) allele displays stronger resistance to anticancer agents compared to the 1199G allele [12,19]. The strong effects of the 1199G > A substitution in the present study are consistent with the fact that it changes Ser400 to Asn near the substrate binding site in P-gp [20,21].

Steroid hormones and their metabolites are already known to regulate expression and activity of *ABCB1* (1199G) P-gp, but whether estrogen and progesterone can regulate *ABCB1* (1199A) P-gp is unclear. Here we provide evidence that estriol, ethynyl estradiol, and estrone are substrates of wild-type and variant P-gp. In addition, ethynyl estradiol and progesterone significantly stimulate ATPase activity in both types of P-gp. The present *in vitro* study justifies *in vivo* experiments to elucidate in greater detail how these hormones modulate P-gp expression and activity, and how this modulation is influenced by various *ABCB1* polymorphisms. For example, our results may be relevant to a report linking the *ABCB1* G3435T polymorphism to efficacy of corticoid therapy in patients with Crohn’s disease [22].

In our bidirectional transport assay in parental LLC-PK1, *ABCB1* (1199G)-LLC-PK1 and *ABCB1* (1199A)-LLC-PK1 cells, *ABCB1* (1199A) P-gp gave much higher net efflux ratios for estriol, ethynyl estradiol, and estrone than did *ABCB1* (1199G) P-gp, suggesting that the three hormones are better substrates for the variant protein. This difference between the P-gp proteins reflects, in part, differences in how much their expression is up-regulated by the three hormones. Progesterone and norethindrone, in contrast, do not appear to be substrates for wild-type or variant P-gp. Repeating the transport assays in the presence of the specific P-gp inhibitor GF120918 led to net efflux ratios below 2.0, defined as the lower limit of P-gp transport.

The present work is the first, to our knowledge, that demonstrates several effects of the 1199G > A SNP on P-gp-dependent efflux of steroid sex hormones. In-depth studies must be performed to demonstrate the complex interactions between steroid sex hormones and pleomorphic P-gp, as well as explore how these interactions may affect clinical outcomes. The ability of steroid hormones to induce P-gp expression and transport activity in human tissue needs to be examined further, such as during the menstrual cycle, when changes in hormone levels alter P-gp expression and activity. Greater insights into how hormones influence the pharmacokinetics and P-gp-mediated efflux of drugs may improve our ability to predict complications and poor efficacy and, thereby, personalize treatment, particularly for women on steroid hormone therapy.

## 5. Conclusions

This is the first study to evaluate the effects of the *ABCB1* (1199G > A) polymorphism on steroid sex hormone-induced P-gp expression, ATPase activity, and hormone efflux. Our findings suggest that estrone, estriol, and ethynyl estradiol are all substrates for both two types of P-gp, and these hormones are able to stimulate the expression levels of P-gp proteins and mRNA in vitro. Two types of P-gp ATPase catalytic activity were induced by ethynyl estradiol and progesterone, but also influenced by *ABCB1* (1199G > A) polymorphism. Therefore, it should be widely considered that SNP may be a important factor to affect the pharmacokinetics and efficacy of chemotherapeutics in patients.
